# Exposure to Phthalate, an Endocrine Disrupting Chemical, Alters the First Trimester Placental Methylome and Transcriptome in Women

**DOI:** 10.1038/s41598-018-24505-w

**Published:** 2018-04-17

**Authors:** N. M. Grindler, L. Vanderlinden, R. Karthikraj, K. Kannan, S. Teal, A. J. Polotsky, T. L. Powell, I. V. Yang, T. Jansson

**Affiliations:** 1 Department of OBGYN, Division of Reproductive Endocrinology and Infertility, University of Colorado, Anschutz Medical Campus, 12631 East 17th Avenue, Room 4403, B198-6, Aurora, CO 80045 USA; 2 Department of Biostatistics and Informatics, Colorado School of Public Health, University of Colorado Anschutz Medical Campus, 13001, 17th Place, Mail Stop B119 Room W3129, Building 500, Aurora, CO 80045 USA; 3 Wadsworth Center, New York State Department of Health, Empire State Plaza, P.O. Box 509, Albany, NY 12201-0509 USA; 4 Department of OBGYN, Division of Family Planning, Anschutz Medical Campus, University of Colorado Hospital, 12605 E. 16th Ave., Aurora, CO 80045 USA; 5 Department of Pediatrics, Section of Neonatology, University of Colorado, Anschutz Medical Campus, 12700 E. 19th Avenue, MS 8613, Aurora, CO 80045 USA; 6 Department of OBGYN, Division of Reproductive Sciences, University of Colorado, Anschutz Medical Campus, 12700 E. 19th Avenue, MS 8613, Aurora, CO 80045 USA; 7 Department of Medicine and Integrated Center for Genes, Environment, and Health, University of Colorado, Anschutz Medical Campus, 12700 East 19th Ave., Mail Stop 8617, Research Center Two, Aurora, CO 80045 USA

## Abstract

Phthalates are known endocrine disruptors and associated with decreased fecundity, pregnancy loss, and adverse obstetrical outcomes, however the underlying mechanisms remain to be established. Environmental factors can influence gene expression and cell function by modifying epigenetic marks, impacting the developing embryo as well as future generations of offspring. The impact of phthalates on placental gene methylation and expression is largely unknown. We studied the effect of maternal phthalate exposure on the human placental DNA methylome and transcriptome. We determined epigenome-wide DNA methylation marks (Illumina Infinium Human Methylation 850k BeadChip) and gene expression (Agilent whole human genome array) associated with phthalate exposure in first trimester placenta. Integrative genomic analysis of candidate genes was performed to define gene methylation-expression relationships. We identified 39 genes with significantly altered methylation and gene expression in the high phthalate exposure group. Most of these relationships were inversely correlated. This analysis identified epidermal growth factor receptor (EGFR) as a critical candidate gene mediating the effects of phthalates on early placental function. Although additional studies are needed to determine the functional consequences of these changes, our findings are consistent with the model that phthalates impact placental function by modulating the expression of critical placental genes through epigenetic regulation.

## Introduction

Around the world pregnant women are exposed to hundreds of different chemicals, however the effects of many of these compounds on human biology remain unexplored. Endocrine-disrupting chemicals (EDCs), which may interfere with any aspect of *in vivo* hormonal action, are of particular concern^1^. Phthalates are a ubiquitous type of plasticizers used in a wide range of consumer products including toys, food packaging, cosmetic products, and medical equipment. One of the most common phthalates, di(2-ethylhexyl) phthalate (DEHP), represents a particular public health concern because 100% of the US population have measurable levels of this EDC^2^. We have previously reported that DEHP is associated with significantly earlier menopause in women, suggesting that DEHP negatively impacts reproduction^1^. Exposure to phthalates is also associated with decreased couple fecundity, low birth weight, preterm birth, and pregnancy loss^1,3,4^. The placenta is continuously exposed to phthalates throughout pregnancy and this EDC also crosses the placental barrier, raising concerns about impact of this chemical on placental and fetal development^5,6^.

An adverse intrauterine environment has a profound negative influence on the future health of an infant by increasing the risk to develop diseases such as obesity, cardiovascular disease, and cancer later in life^7–10^. The placenta supplies the growing fetus with oxygen and nutrients and functions as an endocrine organ, producing an array of hormones and signaling molecules that adapt maternal physiology during pregnancy and regulate fetal organ development. Placental dysfunction contributes to the development of important pregnancy complications such as preeclampsia and intrauterine growth restriction (IUGR). Thus, any exposure that affects placental function has the potential to alter fetal development and lead to poor health later in life. The impact of EDCs such as phthalates on placental function is largely unknown.

Epigenetic modifications, such as DNA methylation, due to in utero exposures may play a critical role in early programming of disease^11^. Epigenetic mechanisms regulate gene expression by determining the accessibility of DNA for transcriptional processing. DNA methylation is due to methyl groups being added to cytosine residues in DNA regions called CpG sites. When methylation occurs in the promoter region it often leads to transcriptional repression but other more distant regulatory elements, enhancers, are also important in regulation of gene expression^12^. DNA methylation is important for both placental and embryonic development as loss of de novo DNA methyltransferase isoforms 3A and B can result in impaired placental development and early embryonic death^13^.

Genomic imprinting is a special case of epigenetic regulation where genes are repressed (imprinted) by methylation according to the parent-of-origin allele. Genomic imprinting is critical in placental biology, as alterations in the expression of these genes have been linked to placental dysfunction, pregnancy complications, and diseases later in life. Emerging evidence suggests that epigenetic regulation of the placental transcriptome is important for regulating placental growth and differentiation.

Environmental factors can profoundly alter epigenetic marks, impacting the developing embryo as well as the next generation of offspring. Placental genetic and epigenetic profiles may serve as markers environmental exposures^14–16^. For example, loss of placental imprinting at IGF2 has been associated with reduced placental weight and IUGR^17^. Similarly, altered methylation has been linked to abnormal placental morphology and pre-term birth^18^. Intrauterine exposure to the EDC bisphenol A (BPA) markedly alters the methylation of imprinted genes in the mouse placenta^6^, consistent with the possibility that EDCs can alter placental function^19^. Studies examining the impact of environmental exposures on DNA methylation have often focused on the global methylation, using various approaches including the examination of repetitive elements as surrogate markers of global methylation^20^. Specifically, phthalate metabolite levels are inversely correlated to birth weight and positively associated with the methylation of LINE-1 and IGF-2 in term placenta^21,22^. This finding is consistent with the possibility that methylation of these repetitive elements may link prenatal phthalate exposure to IUGR, however epigenetic alterations leading to placental dysfunction are likely to occur much earlier in pregnancy and studies of the impact of phthalates on the first trimester placenta may be more biologically relevant.

EDCs including phthalates are risk factors for adverse health outcomes, but the underlying mechanisms are unclear. Thus, we investigated the relationship between maternal total phthalate (TP) levels and both the expression and methylation profile of placental genes in early human pregnancy. We hypothesized that prenatal phthalate exposure leads to altered placental transcriptome due to changes in placental DNA methylation.

## Methods

### Ethics statement/Study population

**Figure 1:**
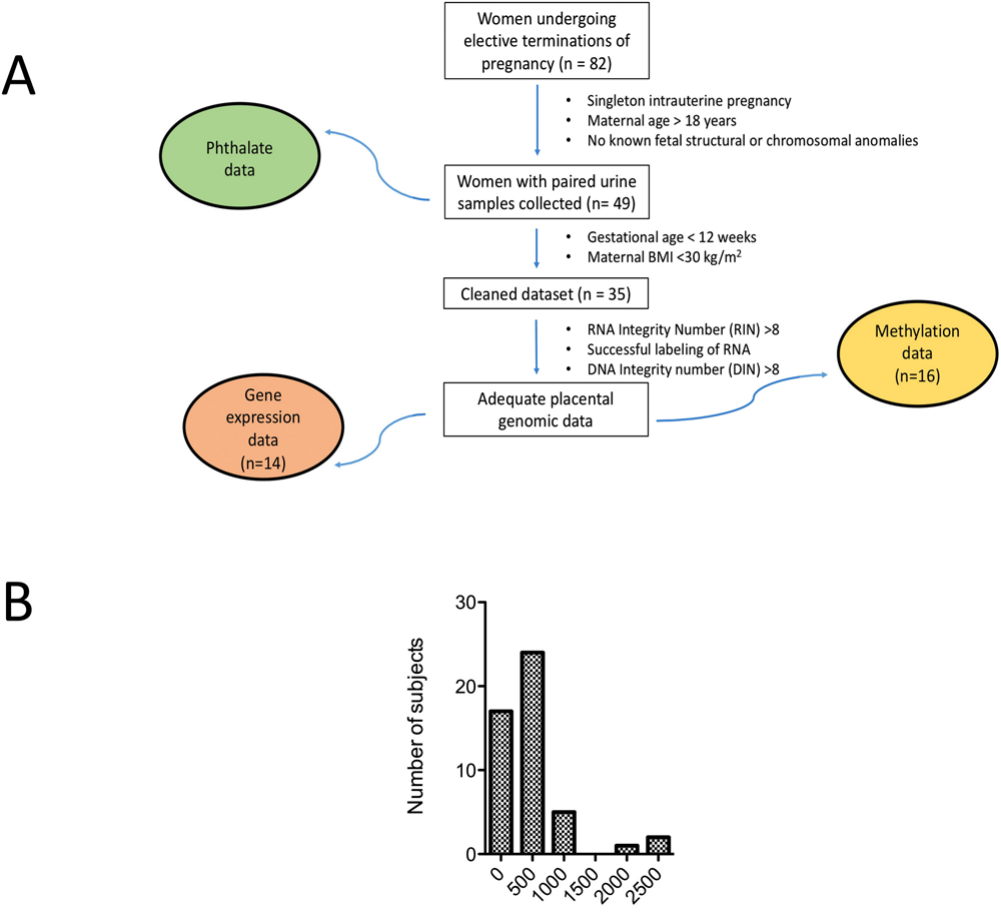
(**A**) Study Design. (**B**) Histogram of total phthalate urinary metabolites in study cohort.

All procedures were approved by the Colorado Multiple Institutional Review Board; all experiments were performed in accordance with relevant guidelines and regulations. De-identified samples and medical information were obtained after informed written consent. Our study population consisted of women undergoing elective terminations in an outpatient setting at the Division of Family Planning, Department of Obstetrics & Gynecology at the University of Colorado during the period June 2015-March 2016. Study participant allocation is shown in Fig. 1 and study participant characteristics are provided in Table 1.

**Table 1:** Demographic characteristics of participants.

	Phthalate Data	Cleaned Dataset	Expression Data	Methylation Data
n	49	34	14	16
Maternal Age (years)	27.7 (19–41)	27.2 (19–41)	29.6 (20–41) p = 0.0565	28.2 (20–41) p = 0.422
Gravidity	3	2	3	3
Parity	1	1	1	1
Body Mass Index (kg/m^2^)	25.2 (16.7–39.5)	22.8 (16.7–28.2)	24 (17.7–28.2) p = 0.467	23.4 (17.7–28.2) p = 0.352
Gestational Age (weeks)	8.71 (5.71–19.4)	7.73 (5.71–11.9)	7.64 (6.14–10.6)	7.64 (6.14–10.6)
**Social Habits #/total (%)**				
Tobacco Use	12/49 (25%)	6/35 (17%)	4/14 (29%)	4/16 (25%)
Alcohol Use	16/49 (32%)	14/35 (40%)	8/14 (57%)	9/16 (56%)
IV drug Use	2/49 (4%)	2/35 (5%)	1/14 (7%)	1/16 (6%)
Marijuana Use	2/49 (4%)	2/35 (5%)	1/14 (7%)	1/16 (6%)

All values are listed as mean (range) unless otherwise specified. ANOVA was performed to assess statistically significant differences as compared to the phthalate data group.

### Sample collection

Maternal urine samples were collected prior to any medical treatment (n = 49) as a spot urine sample^23^. Each sample was analyzed for specific gravity with a refractometer (ATAGO PAL-10S) and frozen at −80 °C. Placental villous tissue was obtained immediately after completion of the procedure, flash frozen, and stored at −80 °C until analysis.

### Measurement of urinary phthalate

Urine samples were shipped to Wadsworth Center (Albany, NY, USA) for quantification of urinary phthalate metabolites concentrations using solid phase extraction coupled with high performance liquid chromatography-isotope dilution tandem mass spectrometry^24^. The chromatographic separation of phthalate metabolites was accomplished using an Agilent 1260 Series HPLC system (Agilent Technologies). Identification and quantification of 23 phthalate metabolites were performed with an ABSCIEX 4500 QTRAP mass spectrometer (Applied Biosystems) under negative ionization mode.

### Analysis of urinary phthalate data

Urinary phthalate metabolite concentrations were adjusted for urinary dilution for each sample. The geometric mean of the concentration of each urinary metabolite was calculated. Values below the limit of detection (LOD) were assigned the value of LOD divided by the square root of 2. Quartiles of distribution were used to identify groups with low and high urine concentrations of total phthalate (TP), respectively. Spearman correlation was used to assess the dependence of each metabolite on demographic parameters.

### Genomic DNA methylation profiling

Genomic DNA was isolated using the Qiagen AllPrep DNA/RNA Mini kit. DNA integrity was established as DNA Integrity Number (DIN) >8. To measure methylation at 853,307 single CpG sites across the genome, we used Illumina’s Infinium Human Methylation 850k BeadChip. 0.85–1.00 µg DNA were bisulfite converted using the Zymo EZ DNA Methylation kit. The labeling, hybridization, and scanning procedures were performed on the iScan system (University of Colorado Genomics Core). This analysis was performed in placental samples obtained from the quartile of women with highest (n = 7) and the lowest TP exposure (n = 9). All samples were assayed once (no technical replicates) with 2 arrays (8 samples per array) performed in 1 batch.

The BeadChips were examined for quality on both the probe and sample level using the minfi package (v1.18.6)^25^ (v3.3.1). We removed probes which failed to be detected above background or probes with low bead count (<3) in more than 10% of our samples as well as probes identified as cross-reactive, resulting in a dataset consistent of 834,015 CpG probes. Data was then normalized using subset-quantile within array normalization (SWAN)^26^. Normalized M-values were used for all subsequent statistical analyses, while β-values (scale of 0–100% methylated) are used for biological interpretation in tables and figures.

### Genomic gene expression profiling

RNA was isolated using the Qiagen AllPrep DNA/RNA Mini kit. RNA integrity was established as RNA Integrity Number (RIN) >8. Total RNA was reverse-transcribed into single-stranded cDNA and then cRNA using random primers in the One-Color Microarray-Based Low Input Quick Amp Labeling (Agilent). Gene expression profiling was performed using microarray (Agilent Sureprint G3 8 × 60 K array).

### Analysis of DNA methylation and gene expression– overall strategy

We identified DNA methylation changes associated with TP level for both single CpG motifs (differentially methylated probes [DMPs]) and differentially methylated regions (DMRs) to be as biologically inclusive as possible. We evaluated the correlations between candidate CpG sites and the corresponding gene expression level from RNA microarrays in order to identify genes affected by methylation. Principal component analysis of methylation data revealed that maternal age, gravidity, parity, body mass index, alcohol use, tobacco use, marijuana use, and intravenous drug use were not significant variables (p < 0.05) and were not included as covariates in our study.

### Statistical analyses of DNA methylation data

An epigenome-wide association analysis was performed to find DMPs between high and low TP groups by performing a t-test using R (lm function, R version 3.3.1) on each CpG probe on the array. A DMR analysis between high and low TP groups was also performed using DMRcate (Version 1.8.6). Due to a large amount of probes tested (834,015) and small sample size, we considered a nominal p-value of 0.005 as significant in both the DMP and DMR analysis. Enrichment analysis using gene symbols from the candidate DMPs and probes present in DMRs (nominal p-value < 0.005) was performed in Panther (Version 11.1) to identify key pathways (Panther Pathways v 3.4.1) and biological processes (Gene ontology, GO database version 1.2). Network Analyst was performed to characterize the methylation-gene expression changes.

### Statistical analysis of gene expression data

Intensity data from the Agilent Feature Extraction were log_2_ transformed and normalized by using RMA with the oligo R package. Differential gene expression was determined using t-test, implemented in the R package limma, and a cut-off of p < 0.005 between high and low total phthalate groups with a 1.5-fold difference in expression. Network Analyst was used for pathway analysis.

## Results

The characteristics of the study subjects included in the different analyses were similar (Table 1). We successfully measured 23 phthalate metabolites in urine (Table 2). The mean TP concentration was 231 ng/mL; the upper quartile for TP was 331 ng/mL (Fig. 1). There was no correlation between TP levels and maternal age, maternal BMI, or gestational age. We compared our results to published results for females in a large cross-sectional cohort found in the United States through the National Health and Nutrition Examination Survey (NHANES). We evaluated 13 additional phthalate metabolites that were not measured in NHANES. Our results were comparable to prior results with the following exceptions: 6 metabolites were lower (mCIOP, mCPP, mEHHP, mECPP, mEOHP, mEHP) and 6 metabolites had higher levels (mBzP, mCHP, mOP, mIBP, mCINP, mBP) in our population compared to NHANES.

**Table 2:** Summary of phthalate data for all 23 metabolites measured.

	Mean (ng/mL)	Geometric Confidence Interval (ng/mL)	NHANES Mean (ng/mL)	NHANES Confidence Interval (ng/mL)
mMP	5.48	4.29–7.01	N/A*	N/A*
mEP	41.4	29.1–58.8	37.7	30.6–46.4
mCPP	0.941	0.671–1.32	2.58	2.24–2.97
PA	34.1	26.3–44.3	N/A	N/A
mEHHP	2.00	1.61–2.47	7.2	6.77–7.66
mBzP	9.35	7.19–12.2	4.27	3.81–4.77
mCHP	<LOD	<LOD	<LOD*	N/A*
mOP	0.207	0.108–0.396	<LOD	N/A
mIDP	12.9	7.89–21.2	N/A	N/A
mHxP	0.171	0.121–0.243	N/A	N/A
mHpP	0.415	0.258–0.667	N/A	N/A
mCIOP	2.8	1.55–3.56	17.1	14.7–19.9
mCINP	4.67	2.68–8.13	2.21	2.03–2.40
mPeP	0.0251	0.0131–0.0483	N/A	N/A
mIPrP	0.0671	0.0438–0.103	N/A	N/A
mBP	9.91	8.09–12.1	7.14	6.05–8.41
mIBP	6.84	5.77–8.13	5.52	4.95–6.15
mECPP	9.42	7.90–11.2	11.8	10.8–12.9
mCMHP	8.54	7.22–10.1	N/A	N/A
mCHpP	0.888	0.581–1.36	N/A	N/A
mEOHP	0.711	0.546–0.926	4.71	4.39–5.07
mINP	2.38	0.923–6.14	N/A*	N/A*
mEHP	0.858	0.622–1.18	1.24	1.14–1.34
Total	230.84	287–455	N/A	N/A

^*^Data not determined for 2011–2012. Data presented is from 1999–2010.

All data reported as mean is geometric mean with corresponding geometric 95% confidence interval. Abbreviations: limit of detection (LOD), mono-(2-ethyl-5-carboxypentyl) phthalate (mECPP), mono-[(2-carboxymethyl) hexyl] phthalate, (mCMHP), mono-(2-ethyl-5-oxohexyl) phthalate (mEOHP), mono-(2-ethyl-5-hydroxyhexyl) phthalate (mEHHP), mono-(3-carboxypropyl) phthalate (mCPP), mono-2-isobutyl phthalate (mIBP), mono-cyclohexyl phthalate (mCHP), mono-isononyl phthalate, (mINP), phthalic acid (PA), mono-(8-methyl-1-nonyl) phthalate (mIDP), mono-octyl phthalate (mOP), mono-n-butyl phthalate (mBP), mono-hexyl phthalate (mHxP), mono-2-heptyl phthalate (mHpP), mono-methyl phthalate (mMP), mono-ethyl phthalate (mEP), mono-benzyl phthalate (mBzP), mono(7-carboxyheptyl)phthalate (mCHpP), mono-isopropyl phthalate (mIPrP), mono-pentyl phthalate (mPeP), mono-carboxy isooctyl phthalate (mCIOP), mono-carboxy isononyl phthalate (mCINP) and mono-ethylhexyl phthalate (mEHP). Data obtained from https://www.cdc.gov/biomonitoring/pdf/FourthReport_UpdatedTables_Volume1_Jan2017.pdf Data for the following compounds was available in NHANES but not evaluated in our study: MHNCH.

**Figure 2:**
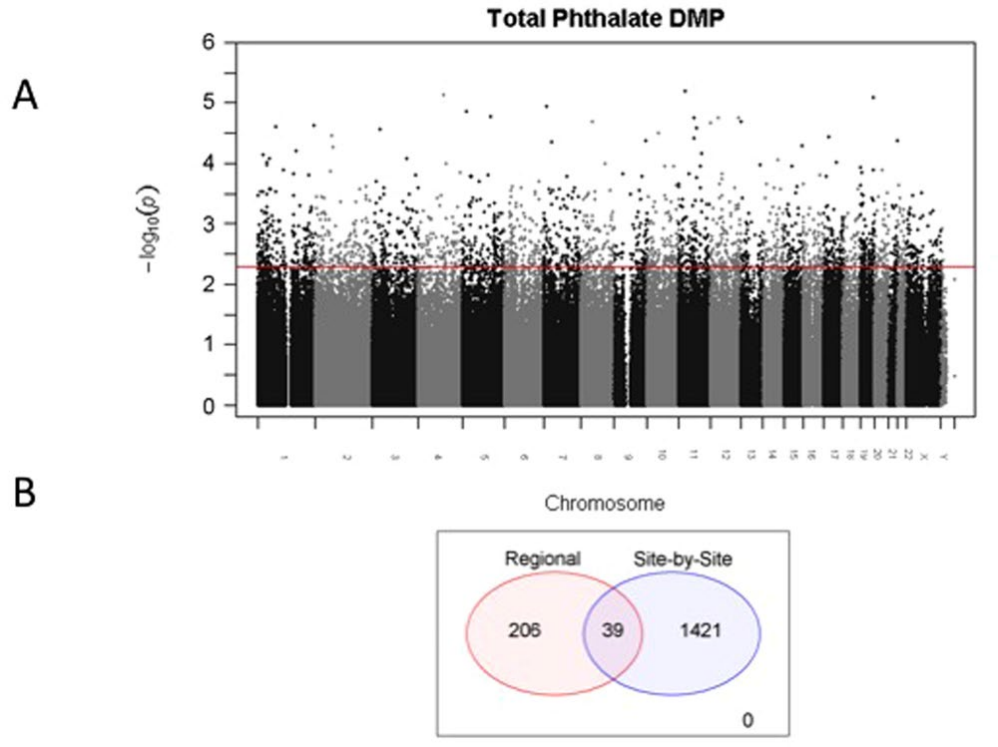
(**A**) Manhattan plot for the DMP analysis. The red line represents the p-value threshold of 0.005 applied to get our candidate list. All CpG probes that lie above this line are considered significant. (**B**) Ven diagram illustrating the overlap of differentially methylated probes (DMP) and differentially methylated region (DMR) analysis.

Given our small sample size, we were unable to perform rigorous adjustment for multiple comparisons when analyzing DNA methylation and gene expression array data but rather examined multiple levels of evidence that DNA methylation/expression of a gene are associated with phthalate exposure. We identified 2214 significantly (p < 0.005) differentially methylated single CpG sites (differentially methylated positions or DMPs), corresponding to 1460 unique genes in early human placenta for high compared to low TP exposure (Fig. 2 and Supplementary Table S1). Regional analysis of DNA methylation data identified 282 significant differentially methylated regions or DMRs (p < 0.005), of which 245 correspond to unique genes (Supplementary Table S2). 39 genes were identified as differentially methylated by both the DMP and DMR analyses (Fig. 2).

We next analyzed gene expression data, focusing only on differentially methylated genes from DMP and DMR analyses. 163 genes of the 1543 tested (represented by 2447 probes on the Agilent array) were differentially expressed (p < 0.005) in the placentas obtained from women in high vs TP groups (Supplementary Figure S1): 124 genes were down-regulated (Supplementary Table S3) and 39 were up-regulated (Supplementary Table S4).

Considering only CpG sites which were identified as candidates in the DMP or DMR analyses (p < 0.005), and correlating those with 2447 expression probes, we performed 5456 Pearson correlations (multiple CpGs per DMRs were considered, explaining the larger number of correlation than 2447 probes on the expression array) between the methylation M-value and the normalized gene expression values. We found 39 significant methylation-gene expression (p < 0.005) correlations, which correspond to 23 unique gene symbols (Supplementary Table S5). Notably, the majority of these relationships are inversely correlated (29 out of 39) and genes containing multiple significant correlations were all in the same direction.

**Figure 3:**
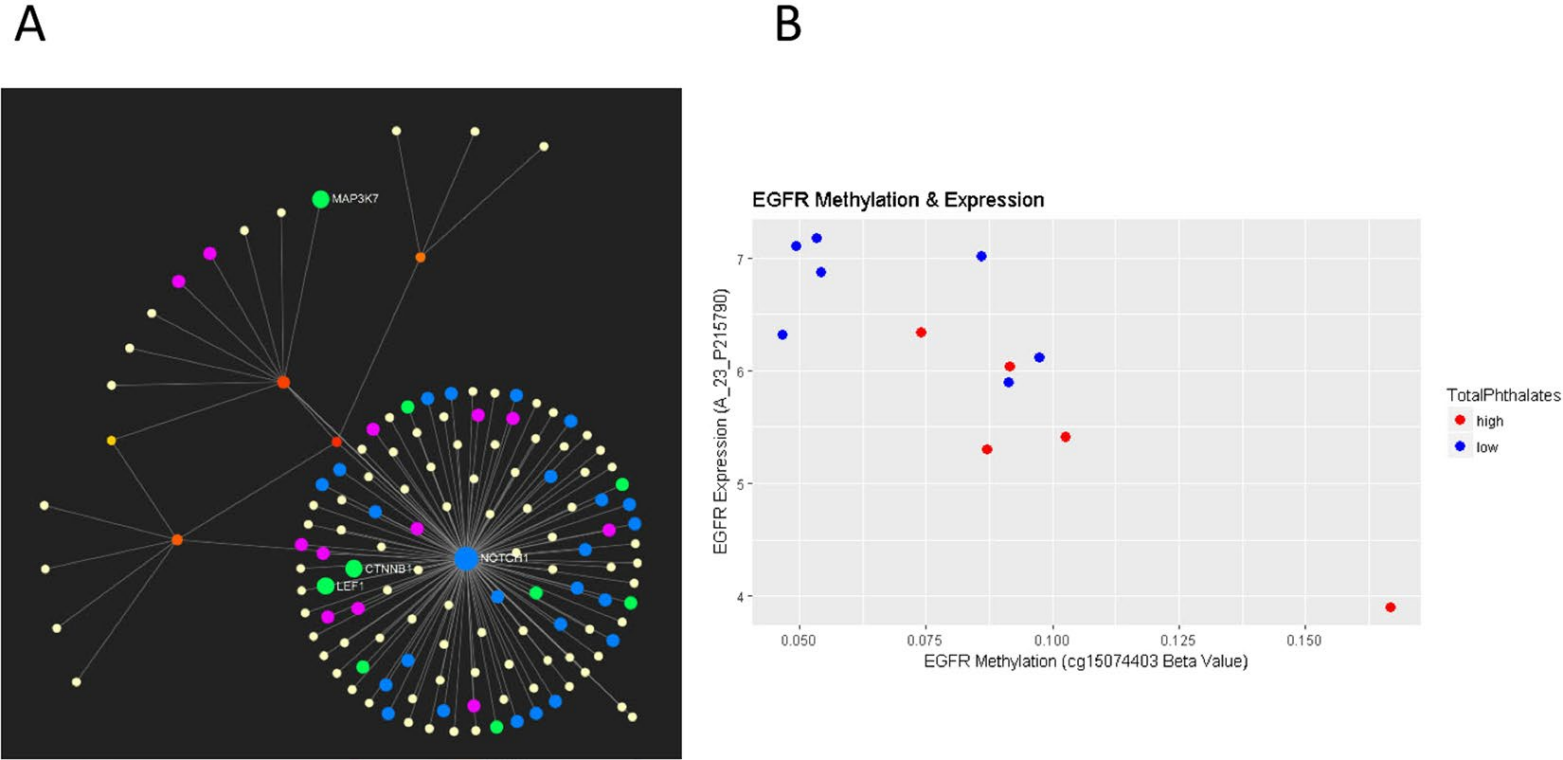
(**A**) Network evaluation resulting from pathway analysis of methylation-gene expression analysis. (**B**) Correlation between methylation and expression for EGFR. The EGFR expression by EGFR methylation levels are plotted. The methylation is represented as Beta-value from the cg15074403 probe while the expression value is represented by probeset A_23_P215790. The Pearson correlation between this expression probeset and the cg15074403 M-value is −0.83 (p-value = 0.00076). Subjects with high phthalate exposure are shown in red, while those with low exposure are shown in blue.

**Figure 4:**
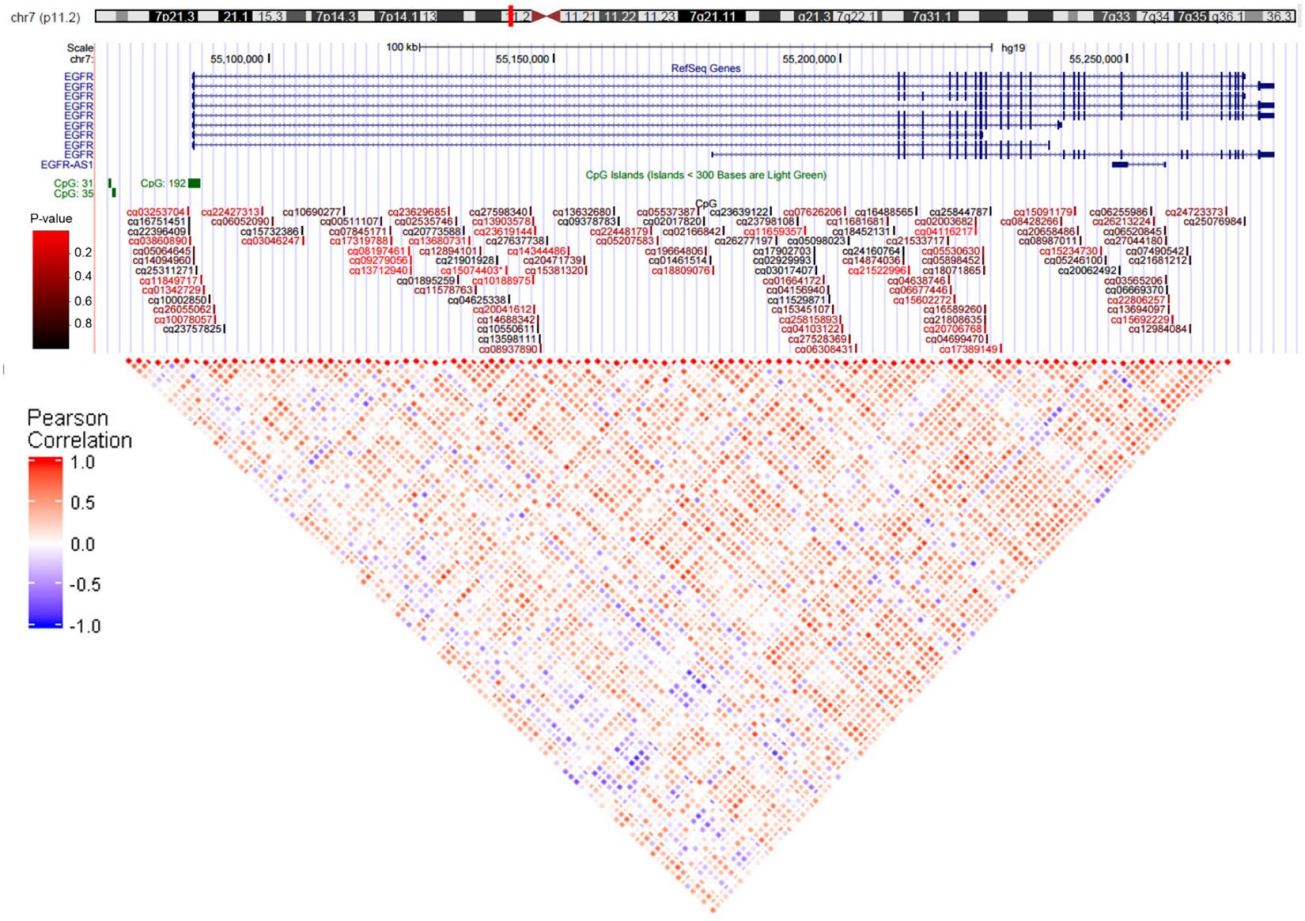
Location of CpG probes in EGFR. The colors of the probes reflect the significance and range from bright red (p-value < 0.005) to black (p-value = 0.99). A heatmap of the Pearson correlation values are shown below and the colors reflect the correlation coefficient with blue representing a coefficient of −1 and red a coefficient of 1.

Pathway analysis of the list of genes identified from methylation-gene expression analysis identified the ErB signaling pathway as the top pathway involved (Supplementary Table S6) (Fig. 3). We determined that *EGFR* was present in 18/51 pathways identified. Notably, *EGFR* was also identified in DMP analysis (Figs 3 and 4).

## Discussion

This is the first study determining the impact of maternal phthalate exposure on human placental gene expression and methylation in early pregnancy. We used a highly reproducible platform that assesses methylation at >850,000 individual CpGs, providing more comprehensive coverage of the epigenome, including recently identified enhancers, than any other study to date in human placenta. We report that phthalate exposure in early pregnancy is associated with alterations in methylation of critical placental genes that are linked to gene expression changes in the expected direction.

The mechanisms underpinning diseases of pregnancy remain poorly understood, possibly because pregnancy complications are multifactorial and involve complex gene-environment interactions that often require large-scale unbiased approaches to interrogate. The rationale to focus on the placenta in early pregnancy is compelling, contributing to the significance of our study. First, the placenta is a formidable endocrine organ, synthesizing and secreting a myriad of hormones into the fetal and maternal circulation, and the placenta may therefore be an early target for endocrine disruption. Second, fetal organogenesis occurs in the first trimester and the fetus is therefore particularly vulnerable during this part of gestation. Third, the placenta is essential for normal fetal development and growth and even subtle changes in placental function in early pregnancy may alter the trajectory for fetal development throughout pregnancy, resulting in long-lasting effects on postnatal health. Fourth, placental gene expression is dynamically regulated across gestation with the first trimester transcriptome being distinct from that of term placenta^27^, highlighting the significance of studying the effects of phthalate exposure on the first trimester placenta. Our findings demonstrate that prenatal exposure to phthalates alters the placental methylome and transcriptome, suggesting that epigenetic mechanisms may link phthalate exposure to changes in placental function and adverse pregnancy outcomes.

Urine phthalate concentrations measured in our study were comparable to a larger sample population of non-pregnant females across the United States. We determined 13 additional phthalate metabolites that were not measured in NHANES, making our study one of the most comprehensive assessments to date of phthalate exposure in early pregnancy. It remains unclear whether the metabolites with significantly different levels are reflective of exposures unique to our population, pregnancy, or a combination of factors. Notably, few epidemiologic studies have determined phthalate levels in pregnancy and little is known about how pregnancy impacts phthalate metabolism in humans. Although some studies have demonstrated changes in the concentrations of urine phthalate metabolites across gestation^28,29^, others have not consistently observed this trend in similar populations^30^. Our data is reflective of our specific population in an urban area but further studies are needed to characterize phthalate levels in normal pregnancy.

Early pregnancy represents a highly orchestrated sequential series of events including fertilization, blastocyst attachment and implantation, uterine decidualization, placentation and fetal development that are regulated by a complex interplay of molecular and cellular regulation. Previous studies have demonstrated that placental genes involved in cell cycle, protein localization, and protein ubiquitination are often highly methylated, while genes in the placenta involved in transcription and developmental function are typically unmethylated^31^. Our report expands on previous epigenome-wide studies evaluations by performing multiple analyses that identified EGFR as a critical candidate gene mediating the effects of phthalates on early placental function.

We found that the placental ErbB signaling was one of the top ranked signaling pathways altered in response to phthalate exposure, both in DNA methylation and correlation of DNA methylation and gene expression, suggesting this critical pathway as a potential target for endocrine disruption. The ErbB signaling pathway includes a receptor tyrosine kinase, that results in the activation of numerous signaling cascades which in turn regulate a wide range of cellular events including proliferation, survival, migration/invasion, or differentiation^32^. Two members of this pathway include epidermal growth factor (EGF) and EGFR, both of which and are critical to placental physiology. EGF stimulates placental growth and function^33,34^ including trophoblast proliferation, differentiation, and invasion^35^. EGFR induces cell cycle progression in placental trophoblasts and has been proposed as a marker for proliferation in trophoblasts^36^. We identified placental EGFR hypermethylation and decreased expression in women with high total phthalate exposure, suggesting that this gene specifically may be a target for endocrine disruption by phthalates.

Dysregulation of epigenetic marks at DMRs in the placenta result in abnormal gene expression leading to major phenotypic changes^37^, and are associated with developmental abnormalities, placental disorders, and malignancies^38^. Epigenetic regulation, in the form of hypermethylation, of the ErbB signaling pathway has previously been implicated in adverse obstetrical outcomes such as preeclampsia^39^. Decreased expression of placental EGFR has been shown to be associated with obstetrical complications^40,41^: placental expression of full-length EGFR mRNA transcript is decreased in IUGR while in preeclampsia, placental and plasma EGF levels are reduced. Additionally, EGFR signaling is crucial in the interaction between the cytotrophoblast and environmental exposures such as tobacco smoke^42^. Given that ErbB signaling, which includes EGF and EGFR, emerged as one of the top ranked signaling pathways altered in response to phthalate exposure, these findings are consistent with an important role of EGFR in mediating the effect of phthalates on placental function. The mechanism by which phthalates interfere with DNA methylation remains unclear. However, phthalates are known to increase the production of reactive oxygen species^43^. Oxidative DNA damage could inhibit binding of methyl-CpG binding proteins and alter DNA methyltransferase function^44^. Further work is needed to fully understand mechanisms that link phthalate exposure to epigenetic placental dysregulation of EGFR.

Phthalate exposure has previously been associated with altered DNA methylation of growth-related genes in the term human placenta. Specifically, urinary mono (2-ethyl-5-hydroxyhexyl) phthalate (MEHHP), and mono (2-ethyl-5-oxyohexyl) phthalate (MEOHP) are inversely associated with placental IGF2 DNA methylation^22^. These associations were determined in growth restricted infants, suggesting that changes in placental DNA methylation represent an underlying biological pathway linking prenatal phthalate exposure and IUGR^22^. Our study did not find significant alterations in methylation patterns at IGF2. It is possible that epigenetic modifications of IGF2 occur later in gestation and were not evident in our first trimester placental samples. Similarly, it is possible that altered imprinting due to phthalate exposure at IGF2 is a unique pathway for IUGR. Previous work in mice demonstrates that exposure to BPA, alters the expression of the imprinted genes in addition to reduced genome-wide methylation in the placenta^6^. These specific imprinted genes were not differentially methylated in our study, suggesting that the mechanisms by which BPA and phthalates modify the placental methylome are distinct^6^.

Considerable strengths of our study include the measurement of EDC exposure during the first trimester, which represents a critical window of exposure for inducing epigenetic changes, and the comprehensive measurement of several phthalates, which is representative of the wide variety of chemicals humans are exposed to simultaneously. A potential limitation of our study was the use of a single time point urine measurement to determine phthalate exposure. However, several prior studies have demonstrated that a single measurement of select phthalate biomarkers remains consistent throughout pregnancy^45,46^. Another potential limitation of this study is that only a subset of samples was used for expression analysis due to our strict inclusion criteria related to genomic integrity. Importantly, however, the subject characteristics in this subset did not differ from the larger group of subjects. In addition, we studied first trimester placenta and the outcomes of these pregnancies had they gone to term are unknown. Finally, our small sample size precluded adjustment for genome-wide significance in DNA methylation data and therefore some of the genes identified in our analyses may be false positives. To mitigate this, we performed both single CpG and regional analyses, and focused mainly on the intersection of the two. We also used correlation with gene expression to further add to the evidence that differential methylation of a specific gene is associated with phthalate exposure. The effect size of our findings is relatively small, however, this is quite a common finding in environmental epigenetic studies (Breton *et al*. 2017). Early-life exposures typically produce relatively small effects on DNA methylations while large magnitude epigenetic effect sizes may be incompatible with continued development. Thus, we maximized data reliability via stringent quality control and data processing procedures.

Despite these potential limitations, our study identified substantial and consistent first trimester DNA methylation changes associated to phthalate exposures. These methylation changes are associated with gene expression changes, in particular for *EGFR*, and need to be further evaluated in larger cohorts for their potential role as mechanistic links, biomarkers, and therapeutic targets. Confirmation and expansion of these findings in other populations, with additional environmental data as well as studies of the mechanistic basis for altered DNA methylation, will provide valuable insights into the epigenetic mechanisms modulating fetal development. Although future studies are needed to determine the functional consequences of these changes in gene expression, our report is consistent with the model that phthalates impact placental function by modulating the expression of critical placental genes through epigenetic regulation. In humans, this sensitive developmental window coincides with the earliest age of pregnancy at a time when it is not yet clinically recognized. Thus, to prevent adverse effects of phthalate exposure on pregnancy outcome and infant long-term outcomes, exposure management should begin even before a woman gets pregnant.

## Electronic supplementary material

Supplementary information

Table S1

Table S2

Table S5
