# Supplementary material for: Exposure to Phthalate, an Endocrine Disrupting Chemical, Alters the First Trimester Placental Methylome and Transcriptome in Women
Source: Sci Rep. 2018 Apr 17;8:6086. doi: 10.1038/s41598-018-24505-w (PMC5904105; doi:10.1038/s41598-018-24505-w)

**EXPOSURE TO PHTHALATE, AN ENDOCRINE DISRUPTING CHEMICAL, ALTERS THE FIRST TRIMESTER PLACENTAL METHYLOME AND TRANSCRIPTOME IN WOMEN**

N. M. Grindler,^1^ L. Vanderlinden,^2^ R. Karthikraj,^3^ K. Kannan,^3^ S. Teal,^4^ A. J. Polotsky,^1^ T. L. Powell,^5,6^ I.V Yang^7*^ and T. Jansson^6*^

^1^Department of OBGYN, Division of Reproductive Endocrinology and Infertility, University of Colorado, Anschutz Medical Campus, 12631 East 17th Avenue, Room 4403, B198-6, Aurora, CO 80045

^2^ Department of Biostatistics and Informatics, Colorado School of Public Health, University of Colorado Anschutz Medical Campus, 13001 17th Place, Mail Stop B119 Room W3129, Building 500, Aurora, CO 80045

^3^Wadsworth Center, New York State Department of Health, Empire State Plaza, P.O. Box 509, Albany, NY 12201-0509

^4^ Department of OBGYN, Division of Family Planning, Anschutz Medical Campus, University of Colorado Hospital, 12605 E. 16th Ave., Aurora, CO 80045

^5^ Department of Pediatrics, Section of Neonatology, University of Colorado, Anschutz Medical Campus, 12700 E. 19th Avenue, MS 8613, Aurora, CO 80045

^6^ Department of OBGYN, Division of Reproductive Sciences, University of Colorado, Anschutz Medical Campus, 12700 E. 19th Avenue, MS 8613 Aurora, CO 80045

^7^ Department of Medicine and Integrated Center for Genes, Environment, and Health, University of Colorado, Anschutz Medical Campus, 12700 East 19th Ave., Mail Stop 8617, Research Center Two, Aurora, CO 80045

*Shared senior authors

KEYWORDS: Phthalates, epigenetics, endocrine disruptors, pregnancy, pregnancy complications, reproduction, birth outcomes

SUPPORT: NIH T32 HD040135-13 (NMG), UCD OBGYN Academic Enrichment Fund (NMG)

Corresponding Author:

Natalia M Grindler, MD

Department of Obstetrics and Gynecology

University of Colorado, Anschutz Medical Campus

12631 E 17^th^ Ave

Aurora, CO 80045

(303) 724-2014

(303) 724-2061 (FAX)

[ngrindler@gmail.com](mailto:ngrindler@gmail.com)

**Supplementary Table of Contents**

SUPPLEMENTARY TABLE S1. Differentially methylated probes (DMPs) in the placenta identified in the comparison of high and low total phthalate exposure. A). DMP summary. B.) DMP individual probes

(See attached Excel file)

SUPPLEMENTARY TABLE S2. Differentially methylated regions (DMRs) in the placenta identified in the comparison of high and low total phthalate exposure. A). DMR summary. B.) DMR individual probes

(See attached Excel file)

SUPPLEMENTARY TABLE S3. Down-regulated placental genes in study subjects with high phthalate exposure compared to those with low total phthalate exposure in early pregnancy. Genes listed as “Unknown” are probes that are not currently associated with a known gene and/or gene function.

SUPPLEMENTARY TABLE S4. Up-regulated placental genes in study subjects with high total phthalate exposure compared to those with low total phthalate exposure in early pregnancy. Genes listed as “Unknown” are probes that are not currently associated with a known gene and/or gene function.

SUPPLEMENTARY TABLE S5. List of genes identified in the methylation-correlation analysis.

(See attached Excel file)

SUPPLEMENTARY TABLE S6. List of enrichment results from Network Analyst for genes identified in methylation-gene expression analysis.

SUPPLEMENTARY FIGURE S1. Gene expression stratification between high and low total phthalate groups. Red groups represent high maternal total phthalate levels. Blue groups represent low total maternal phthalate level.

SUPPLEMENTARY TABLE S3. Down-regulated placental genes in study subjects with high phthalate exposure compared to those with low total phthalate exposure in early pregnancy. Genes listed as “Unknown” are probes that are not currently associated with a known gene and/or gene function.

| **Probeset ID** | **Gene Name** | **Gene Symbol** | **Fold-Change** | **p-value** |
| --- | --- | --- | --- | --- |
| A_21_P0000622 | WDR86 antisense RNA 1 | WDR86-AS1 | -16.41 | 0.00468 |
| A_33_P3243332 | Rho GTPase activating protein 42 | ARHGAP42 | -10.99 | 0.00223 |
| A_33_P3387696 | transmembrane BAX inhibitor motif containing 4 | TMBIM4 | -6.39 | 0.00347 |
| A_23_P259393 | Scm-like with four mbt domains 1 | SFMBT1 | -5.77 | 0.00008 |
| A_33_P3268310 | LIM and senescent cell antigen-like domains 3-like | LIMS3L | -4.42 | 0.00043 |
| A_33_P3294881 | coiled-coil domain containing 174 | CCDC174 | -4.32 | 0.00264 |
| A_23_P377434 | trinucleotide repeat containing 18 | TNRC18 | -3.65 | 0.00284 |
| A_33_P3216297 | nuclear receptor subfamily 3, group C, member 1 (glucocorticoid receptor) | NR3C1 | -3.26 | 0.00424 |
| A_23_P31747 | chromosome 8 open reading frame 76 | C8orf76 | -3.10 | 0.00324 |
| A_33_P3214314 | coiled-coil serine-rich protein 2 | CCSER2 | -2.99 | 0.00336 |
| A_21_P0012385 | | XLOC_l2_009804 | -2.40 | 0.00412 |
| A_33_P3224858 | eukaryotic translation initiation factor 2-alpha kinase 2 | EIF2AK2 | -2.06 | 0.00161 |
| A_33_P3359973 | | Unknown | -2.03 | 0.00114 |
| A_33_P3329023 | family with sequence similarity 69, member A | FAM69A | -2.02 | 0.00297 |
| A_33_P3893191 | diazepam binding inhibitor-like 5 pseudogene 2 | DBIL5P2 | -1.96 | 0.00327 |
| A_22_P00014572 | lnc-SLA2-2:1 | lnc-SLA2-2 | -1.94 | 0.00259 |
| A_21_P0003286 | lnc-ADAMTS9-3:2 | lnc-ADAMTS9-3 | -1.94 | 0.00120 |
| A_33_P3408529 | | Unknown | -1.90 | 0.00465 |
| A_21_P0007349 | lnc-CHST1-1:1 | lnc-CHST1-1 | -1.89 | 0.00441 |
| A_33_P3243163 | | Unknown | -1.88 | 0.00249 |
| A_22_P00016560 | uncharacterized LOC101927513 | LOC101927513 | -1.88 | 0.00384 |
| A_22_P00024134 | lnc-SIAH1-1:1 | lnc-SIAH1-1 | -1.88 | 0.00398 |
| A_33_P3323535 | | Unknown | -1.88 | 0.00422 |
| A_22_P00023236 | lnc-RAB9B-1:1 | lnc-RAB9B-1 | -1.87 | 0.00490 |
| A_22_P00013855 | lnc-RPL7-1:3 | lnc-RPL7-1 | -1.87 | 0.00363 |
| A_23_P3911 | plexin domain containing 1 | PLXDC1 | -1.87 | 0.00472 |
| A_21_P0007565 | | Unknown | -1.87 | 0.00424 |
| A_21_P0009766 | lnc-NFKBID-1:1 | lnc-NFKBID-1 | -1.87 | 0.00357 |
| A_21_P0005559 | lnc-RAMP3-2:1 | lnc-RAMP3-2 | -1.87 | 0.00354 |
| A_21_P0002676 | lnc-MKI67IP-1:3 | lnc-MKI67IP-1 | -1.86 | 0.00422 |
| A_21_P0010029 | lnc-FAM182B-1:1 | lnc-FAM182B-1 | -1.85 | 0.00174 |
| A_33_P3298206 | protein phosphatase 4, regulatory subunit 4 | PPP4R4 | -1.85 | 0.00473 |
| A_23_P97112 | selectin E | SELE | -1.85 | 0.00283 |
| A_21_P0012027 | testis expressed 41 (non-protein coding) | TEX41 | -1.85 | 0.00360 |
| A_23_P49842 | unc-119 homolog (C. elegans) | UNC119 | -1.85 | 0.00426 |
| A_23_P166910 | DnaJ (Hsp40) homolog, subfamily C, member 13 | DNAJC13 | -1.85 | 0.00031 |
| A_33_P3236133 | SHQ1, H/ACA ribonucleoprotein assembly factor | SHQ1 | -1.84 | 0.00303 |
| A_21_P0008036 | lnc-TNFRSF19-5:7 | lnc-TNFRSF19-5 | -1.84 | 0.00278 |
| A_21_P0012504 | | Unknown | -1.84 | 0.00373 |
| A_24_P339560 | sialic acid binding Ig-like lectin 11 | SIGLEC11 | -1.83 | 0.00285 |
| A_33_P3307013 | EF-hand calcium binding domain 13 | EFCAB13 | -1.83 | 0.00437 |
| A_22_P00021400 | lnc-MAFK-2:2 | lnc-MAFK-2 | -1.83 | 0.00485 |
| A_23_P208302 | apolipoprotein C-II | APOC2 | -1.83 | 0.00298 |
| A_24_P6428 | family with sequence similarity 217, member B | FAM217B | -1.83 | 0.00358 |
| A_33_P3365553 | long intergenic non-protein coding RNA 937 | LINC00937 | -1.83 | 0.00143 |
| A_33_P3407007 | long intergenic non-protein coding RNA 861 | LINC00861 | -1.82 | 0.00381 |
| A_23_P428298 | unc-5 homolog C (C. elegans)-like | UNC5CL | -1.81 | 0.00204 |
| A_21_P0002234 | lnc-ETAA1-6:1 | lnc-ETAA1-6 | -1.81 | 0.00203 |
| A_33_P6811736 | uncharacterized LOC643770 | LOC643770 | -1.81 | 0.00204 |
| A_21_P0000376 | small nucleolar RNA, C/D box 32B | SNORD32B | -1.81 | 0.00349 |
| A_33_P3251369 | GRB2-associated binding protein 3 | GAB3 | -1.81 | 0.00408 |
| A_33_P3219960 | | Unknown | -1.80 | 0.00039 |
| A_22_P00010661 | lnc-NELL1-1:1 | lnc-NELL1-1 | -1.80 | 0.00434 |
| A_21_P0007864 | uncharacterized LOC101927694 | LOC101927694 | -1.80 | 0.00248 |
| A_24_P417352 | uncharacterized LOC102725284 | LOC102725284 | -1.80 | 0.00214 |
| A_33_P3264188 | olfactory receptor, family 2, subfamily D, member 3 | OR2D3 | -1.80 | 0.00218 |
| A_21_P0007249 | RAB30 antisense RNA 1 (head to head) | RAB30-AS1 | -1.80 | 0.00443 |
| A_24_P573533 | COBW domain containing 5 | CBWD5 | -1.79 | 0.00225 |
| A_33_P3283147 | olfactory receptor, family 5, subfamily M, member 11 | OR5M11 | -1.79 | 0.00309 |
| A_33_P3395630 | | Unknown | -1.79 | 0.00230 |
| A_22_P00013447 | uncharacterized LOC102724966 | LOC102724966 | -1.79 | 0.00473 |
| A_21_P0005602 | lnc-RP4-725G10.1.1-5:2 | lnc-RP4-725G10.1.1-5 | -1.78 | 0.00355 |
| A_23_P301304 | fibroblast growth factor receptor 1 | FGFR1 | -1.78 | 0.00295 |
| A_32_P129894 | multiple EGF-like-domains 9 | MEGF9 | -1.78 | 0.00490 |
| A_21_P0012934 | | XLOC_l2_012323 | -1.78 | 0.00490 |
| A_21_P0010828 | | XLOC_l2_001592 | -1.78 | 0.00161 |
| A_33_P3301291 | forkhead box B2 | FOXB2 | -1.78 | 0.00191 |
| A_21_P0004367 | lnc-CDH18-4:1 | lnc-CDH18-4 | -1.78 | 0.00247 |
| A_33_P3414789 | fibronectin type III and SPRY domain containing 1 | FSD1 | -1.78 | 0.00320 |
| A_21_P0006904 | lnc-SORCS3-3:1 | lnc-SORCS3-3 | -1.77 | 0.00152 |
| A_22_P00016331 | lnc-TMEM156-1:1 | lnc-TMEM156-1 | -1.77 | 0.00214 |
| A_21_P0001845 | uncharacterized LOC102723362 | LOC102723362 | -1.77 | 0.00147 |
| A_22_P00020246 | lnc-GPR55-2:2 | lnc-GPR55-2 | -1.77 | 0.00421 |
| A_23_P141863 | zinc finger protein 544 | ZNF544 | -1.77 | 0.00144 |
| A_33_P3284472 | DEAD (Asp-Glu-Ala-Asp) box polypeptide 51 | DDX51 | -1.76 | 0.00499 |
| A_21_P0004417 | RASGRF2 antisense RNA 1 | RASGRF2-AS1 | -1.76 | 0.00274 |
| A_32_P32905 | MYST/Esa1-associated factor 6 | MEAF6 | -1.76 | 0.00396 |
| A_33_P3405504 | | Unknown | -1.75 | 0.00286 |
| A_33_P3224878 | integrin, alpha 4 (antigen CD49D, alpha 4 subunit of VLA-4 receptor) | ITGA4 | -1.75 | 0.00487 |
| A_33_P3332955 | C-type lectin domain family 1, member B | CLEC1B | -1.75 | 0.00148 |
| A_33_P3321324 | uncharacterized LOC642691 | FLJ37786 | -1.75 | 0.00136 |
| A_23_P73012 | chromosome 9 open reading frame 3 | C9orf3 | -1.75 | 0.00167 |
| A_21_P0014790 | | Unknown | -1.74 | 0.00226 |
| A_19_P00322260 | uncharacterized LOC101929709 | LOC101929709 | -1.74 | 0.00139 |
| A_22_P00016527 | | Unknown | -1.74 | 0.00463 |
| A_22_P00008427 | lnc-KANK4-1:1 | lnc-KANK4-1 | -1.73 | 0.00143 |
| A_33_P3333677 | | Unknown | -1.73 | 0.00324 |
| A_23_P86710 | FERM and PDZ domain containing 2 | FRMPD2 | -1.73 | 0.00339 |
| A_19_P00319793 | uncharacterized LOC100287948 | GM140 | -1.73 | 0.00113 |
| A_22_P00015491 | EPHA5 antisense RNA 1 | EPHA5-AS1 | -1.73 | 0.00106 |
| A_21_P0003267 | | Unknown | -1.72 | 0.00368 |
| A_33_P3883116 | CACNA1G antisense RNA 1 | CACNA1G-AS1 | -1.72 | 0.00099 |
| A_33_P3343800 | | Unknown | -1.71 | 0.00094 |
| A_32_P149536 | small ubiquitin-like modifier 2 | SUMO2 | -1.71 | 0.00156 |
| A_21_P0002315 | lnc-GORASP2-1:1 | lnc-GORASP2-1 | -1.71 | 0.00429 |
| A_22_P00005737 | lnc-EMP2-2:1 | lnc-EMP2-2 | -1.70 | 0.00086 |
| A_21_P0010921 | glutamate dehydrogenase 1 pseudogene 7 | GLUD1P7 | -1.70 | 0.00460 |
| A_22_P00015388 | lnc-SRGAP3-1:8 | lnc-SRGAP3-1 | -1.70 | 0.00496 |
| A_23_P145555 | muscular LMNA-interacting protein | MLIP | -1.70 | 0.00085 |
| A_33_P3254708 | Rho GTPase activating protein 40 | ARHGAP40 | -1.69 | 0.00344 |
| A_33_P3238785 | FMR1 antisense RNA 1 | FMR1-AS1 | -1.69 | 0.00084 |
| A_21_P0013393 | uncharacterized LOC102723946 | LOC102723946 | -1.69 | 0.00084 |
| A_21_P0009659 | lnc-SAFB-1:1 | lnc-SAFB-1 | -1.68 | 0.00212 |
| A_21_P0005043 | lnc-GPR63-4:1 | lnc-GPR63-4 | -1.67 | 0.00085 |
| A_21_P0009066 | lnc-MAF-2:1 | lnc-MAF-2 | -1.67 | 0.00086 |
| A_33_P3359183 | acyl-CoA wax alcohol acyltransferase 2 | AWAT2 | -1.66 | 0.00252 |
| A_22_P00025753 | long intergenic non-protein coding RNA 381 | LINC00381 | -1.66 | 0.00089 |
| A_21_P0003012 | lnc-GAP43-9:1 | lnc-GAP43-9 | -1.66 | 0.00271 |
| A_22_P00013422 | | Unknown | -1.66 | 0.00289 |
| A_21_P0008563 | epididymal protein pseudogene | LOC338963 | -1.66 | 0.00174 |
| A_22_P00006500 | lnc-FGF3-4:1 | lnc-FGF3-4 | -1.65 | 0.00094 |
| A_21_P0008261 | lnc-SLITRK6-3:3 | lnc-SLITRK6-3 | -1.65 | 0.00100 |
| A_33_P3294314 | | Unknown | -1.65 | 0.00312 |
| A_33_P3376234 | putative homeodomain transcription factor 1 | PHTF1 | -1.64 | 0.00331 |
| A_22_P00006174 | ITGB2 antisense RNA 1 | ITGB2-AS1 | -1.64 | 0.00104 |
| A_21_P0006540 | lnc-MAGEE2-1:1 | lnc-MAGEE2-1 | -1.64 | 0.00363 |
| A_33_P3271711 | | Unknown | -1.63 | 0.00393 |
| A_22_P00010817 | lnc-NMNAT3-4:1 | lnc-NMNAT3-4 | -1.63 | 0.00112 |
| A_21_P0013942 | | Unknown | -1.63 | 0.00434 |
| A_21_P0006928 | lnc-GPR26-4:3 | lnc-GPR26-4 | -1.63 | 0.00469 |
| A_23_P392402 | glucagon-like peptide 1 receptor | GLP1R | -1.62 | 0.00138 |
| A_22_P00020231 | lnc-CCNE2-1:3 | lnc-CCNE2-1 | -1.60 | 0.00174 |
| A_21_P0014056 | DDB1 and CUL4 associated factor 8 | DCAF8 | -1.60 | 0.00190 |
| A_21_P0002653 | additional sex combs like transcriptional regulator 2 | ASXL2 | -1.58 | 0.00248 |
| A_21_P0000080 | family with sequence similarity 122C | FAM122C | -1.58 | 0.00271 |
| A_22_P00016692 | | Unknown | -1.58 | 0.00201 |
| A_23_P58464 | protocadherin beta 6 | PCDHB6 | -1.58 | 0.00304 |
| A_24_P933908 | glycoprotein (transmembrane) nmb | GPNMB | -1.58 | 0.00334 |
| A_33_P3384284 | suppressor of Ty 20 homolog (S. cerevisiae)-like 1 | SUPT20HL1 | -1.57 | 0.00434 |
| A_23_P63972 | double C2-like domains, gamma, pseudogene | DOC2GP | -1.57 | 0.00367 |
| A_22_P00024174 | ubiquitin specific peptidase 49 | USP49 | -1.57 | 0.00405 |
| A_23_P82795 | long intergenic non-protein coding RNA 208 | LINC00208 | -1.56 | 0.00449 |
| A_23_P425502 | downstream neighbor of SON | DONSON | -1.52 | 0.00456 |
| A_33_P3316786 | dachshund family transcription factor 1 | DACH1 | -1.48 | 0.00331 |
| A_33_P3329949 | annexin A7 | ANXA7 | -1.43 | 0.00271 |
| A_21_P0001115 | lnc-MATN1-3:1 | lnc-MATN1-3 | -1.41 | 0.00352 |
| A_22_P00008347 | long intergenic non-protein coding RNA 649 | LINC00649 | -1.40 | 0.00430 |
| A_24_P34534 | par-3 family cell polarity regulator beta | PARD3B | -1.35 | 0.00470 |
| A_23_P335981 | homeobox C12 | HOXC12 | -1.27 | 0.00328 |
| A_21_P0012731 | lnc-BMP2K-1:4 | lnc-BMP2K-1 | -1.21 | 0.00227 |
| A_22_P00023010 | | Unknown | -1.17 | 0.00232 |
| A_21_P0009604 | uncharacterized LOC101927879 | LOC101927879 | -1.14 | 0.00348 |
| A_21_P0001686 | lnc-IL12RB2-2:5 | lnc-IL12RB2-2 | -1.11 | 0.00306 |
| A_21_P0009759 | lnc-TSHZ3-1:1 | lnc-TSHZ3-1 | -1.07 | 0.00363 |
| A_23_P72387 | actin filament associated protein 1 | AFAP1 | -1.07 | 0.00456 |
| A_21_P0000944 | | Unknown | -1.06 | 0.00356 |
| A_24_P203134 | DDB1 and CUL4 associated factor 12-like 1 | DCAF12L1 | -1.05 | 0.00494 |

SUPPLEMENTARY TABLE S4. Up-regulated placental genes in study subjects with high total phthalate exposure compared to those with low total phthalate exposure in early pregnancy. Genes listed as “Unknown” are probes that are not currently associated with a known gene and/or gene function.

| Probeset ID | Gene Name | Gene Symbol | Fold-Change | p-value |
| --- | --- | --- | --- | --- |
| A_24_P914940 | MEF2B neighbor | MEF2BNB | 14.45 | 0.000601 |
| A_23_P362712 | abhydrolase domain containing 11 | ABHD11 | 13.04 | 0.002827 |
| A_23_P126486 | ciliary rootlet coiled-coil, rootletin pseudogene 2 | CROCCP2 | 10.96 | 0.000937 |
| A_33_P3287883 | uncharacterized LOC100133331 | LOC100133331 | 10.04 | 0.001809 |
| A_33_P3353520 | ring finger protein 225 | RNF225 | 9.95 | 0.002303 |
| A_33_P3227788 | pantothenate kinase 1 | PANK1 | 9.76 | 0.000148 |
| A_23_P54963 | mitochondrial ribosomal protein L38 | MRPL38 | 9.39 | 0.003954 |
| A_23_P96087 | H1 histone family, member X | H1FX | 9.39 | 0.001621 |
| A_33_P3247190 | coiled-coil and C2 domain containing 1B | CC2D1B | 9.24 | 0.003715 |
| A_33_P3249185 | secretion associated, Ras related GTPase 1A | SAR1A | 8.77 | 0.001548 |
| A_33_P3293918 | SH2 domain containing 3C | SH2D3C | 8.54 | 0.000003 |
| A_33_P3278033 | tumor suppressor candidate 1 | TUSC1 | 7.71 | 0.002735 |
| A_23_P34568 | ADP-ribosylhydrolase like 2 | ADPRHL2 | 7.39 | 0.000690 |
| A_23_P212844 | transforming, acidic coiled-coil containing protein 3 | TACC3 | 6.72 | 0.002252 |
| A_23_P33072 | MAF1 homolog (S. cerevisiae) | MAF1 | 6.67 | 0.004424 |
| A_23_P126623 | phosphogluconate dehydrogenase | PGD | 3.86 | 0.003739 |
| A_33_P3519683 | zinc finger and BTB domain containing 8 opposite strand | ZBTB8OS | 2.66 | 0.004319 |
| A_23_P212284 | POC1 centriolar protein A | POC1A | 2.58 | 0.004488 |
| A_33_P3284662 | EP300 interacting inhibitor of differentiation 2 | EID2 | 2.54 | 0.004425 |
| A_33_P3592015 | hyaluronan binding protein 4 | HABP4 | 2.13 | 0.001786 |
| A_24_P343095 | dihydrofolate reductase | DHFR | 1.98 | 0.004881 |
| A_23_P82296 | guanine nucleotide binding protein (G protein), beta polypeptide 2 | GNB2 | 1.79 | 0.001037 |
| A_33_P3369039 | pre-mRNA processing factor 31 | PRPF31 | 1.61 | 0.003060 |
| A_24_P206047 | solute carrier family 25 (mitochondrial carrier; adenine nucleotide translocator), memb | SLC25A4 | 1.59 | 0.001287 |
| A_23_P68899 | thioredoxin 2 | TXN2 | 1.52 | 0.002183 |
| A_23_P125815 | RNA binding motif protein 10 | RBM10 | 1.39 | 0.001909 |
| A_24_P415601 | ribonuclease/angiogenin inhibitor 1 | RNH1 | 1.39 | 0.003341 |

SUPPLEMENTARY TABLE S6. List of enrichment results from Network Analyst for genes identified in methylation-gene expression analysis.

| Pathway | Total number of genes in pathway | Expected | Hits | P.Value | FDR | EGFR present |
| --- | --- | --- | --- | --- | --- | --- |
| ErbB signaling pathway | 87 | 8.92 | 48 | 7.40E-26 | 1.60E-23 | Y |
| Pathways in cancer | 310 | 31.8 | 89 | 2.14E-21 | 2.32E-19 | Y |
| Chronic myeloid leukemia | 73 | 7.49 | 39 | 1.74E-20 | 1.26E-18 | N |
| Prostate cancer | 87 | 8.92 | 37 | 2.68E-15 | 1.45E-13 | Y |
| Neurotrophin signaling pathway | 123 | 12.6 | 43 | 6.10E-14 | 2.65E-12 | N |
| Glioma | 65 | 6.67 | 30 | 8.91E-14 | 3.22E-12 | Y |
| Epstein-Barr virus infection | 91 | 9.33 | 33 | 1.85E-11 | 5.74E-10 | N |
| Adherens junction | 70 | 7.18 | 28 | 4.38E-11 | 1.19E-09 | Y |
| T cell receptor signaling pathway | 98 | 10.1 | 33 | 1.84E-10 | 4.45E-09 | N |
| Non-small cell lung cancer | 52 | 5.33 | 22 | 1.57E-09 | 3.41E-08 | Y |
| Jak-STAT signaling pathway | 99 | 10.2 | 31 | 5.06E-09 | 9.99E-08 | N |
| Acute myeloid leukemia | 57 | 5.85 | 22 | 1.24E-08 | 2.08E-07 | N |
| Fc gamma R-mediated phagocytosis | 97 | 9.95 | 30 | 1.25E-08 | 2.08E-07 | N |
| HTLV-I infection | 199 | 20.4 | 47 | 1.81E-08 | 2.80E-07 | N |
| Focal adhesion | 200 | 20.5 | 47 | 2.14E-08 | 3.10E-07 | Y |
| Cell cycle | 124 | 12.7 | 34 | 3.95E-08 | 5.36E-07 | N |
| Bacterial invasion of epithelial cells | 56 | 5.74 | 21 | 4.86E-08 | 6.20E-07 | N |
| Insulin signaling pathway | 137 | 14.1 | 36 | 5.20E-08 | 6.27E-07 | N |
| Chemokine signaling pathway | 189 | 19.4 | 43 | 2.38E-07 | 2.72E-06 | N |
| Hepatitis C | 100 | 10.3 | 28 | 4.04E-07 | 4.38E-06 | Y |
| Pancreatic cancer | 69 | 7.08 | 22 | 6.40E-07 | 6.61E-06 | Y |
| B cell receptor signaling pathway | 75 | 7.69 | 23 | 7.75E-07 | 7.31E-06 | N |
| Fc epsilon RI signaling pathway | 75 | 7.69 | 23 | 7.75E-07 | 7.31E-06 | N |
| Renal cell carcinoma | 60 | 6.15 | 20 | 9.39E-07 | 8.49E-06 | N |
| Endocytosis | 101 | 10.4 | 27 | 1.78E-06 | 1.54E-05 | Y |
| MAPK signaling pathway | 265 | 27.2 | 51 | 4.21E-06 | 3.51E-05 | Y |
| Transcriptional misregulation in cancer | 19 | 1.95 | 10 | 4.62E-06 | 3.71E-05 | N |
| Osteoclast differentiation | 119 | 12.2 | 29 | 5.64E-06 | 4.17E-05 | N |
| Chagas disease (American trypanosomiasis) | 89 | 9.13 | 24 | 5.71E-06 | 4.17E-05 | N |
| Natural killer cell mediated cytotoxicity | 138 | 14.2 | 32 | 5.76E-06 | 4.17E-05 | N |
| Tuberculosis | 174 | 17.8 | 37 | 9.34E-06 | 6.54E-05 | N |
| Bladder cancer | 29 | 2.97 | 12 | 1.20E-05 | 8.13E-05 | Y |
| VEGF signaling pathway | 76 | 7.79 | 21 | 1.46E-05 | 9.63E-05 | Y |
| Measles | 102 | 10.5 | 25 | 2.26E-05 | 0.000144 | N |
| Epithelial cell signaling in Helicobacter pylori infection | 37 | 3.79 | 13 | 4.16E-05 | 0.000258 | Y |
| GnRH signaling pathway | 94 | 9.64 | 23 | 4.95E-05 | 0.000298 | Y |
| Colorectal cancer | 49 | 5.03 | 15 | 6.82E-05 | 4.00E-04 | N |
| Aldosterone-regulated sodium reabsorption | 34 | 3.49 | 12 | 7.83E-05 | 0.000447 | N |
| Melanoma | 68 | 6.97 | 18 | 0.000111 | 0.000602 | Y |
| Small cell lung cancer | 80 | 8.21 | 20 | 0.000111 | 0.000602 | N |
| Leishmaniasis | 51 | 5.23 | 15 | 0.000114 | 0.000602 | N |
| Toxoplasmosis | 93 | 9.54 | 22 | 0.000124 | 0.00064 | N |
| Amoebiasis | 46 | 4.72 | 14 | 0.000128 | 0.000645 | N |
| Endometrial cancer | 44 | 4.51 | 13 | 0.000309 | 0.00152 | Y |
| Legionellosis | 40 | 4.1 | 12 | 0.000449 | 0.00217 | N |
| Leukocyte transendothelial migration | 108 | 11.1 | 23 | 0.00046 | 0.00217 | N |
| Gap junction | 89 | 9.13 | 20 | 0.000516 | 0.00238 | Y |
| Notch signaling pathway | 47 | 4.82 | 13 | 0.000627 | 0.00278 | N |
| Antigen processing and presentation | 61 | 6.26 | 15 | 0.000956 | 0.00408 | N |
| Progesterone-mediated oocyte maturation | 80 | 8.21 | 18 | 0.000958 | 0.00408 | N |
| Influenza A | 107 | 11 | 22 | 0.001 | 0.00419 | N |

SUPPLEMENTARY FIGURE S1. Gene expression stratification between high and low total phthalate groups. Red groups represent high maternal total phthalate levels. Blue groups represent low total maternal phthalate level.


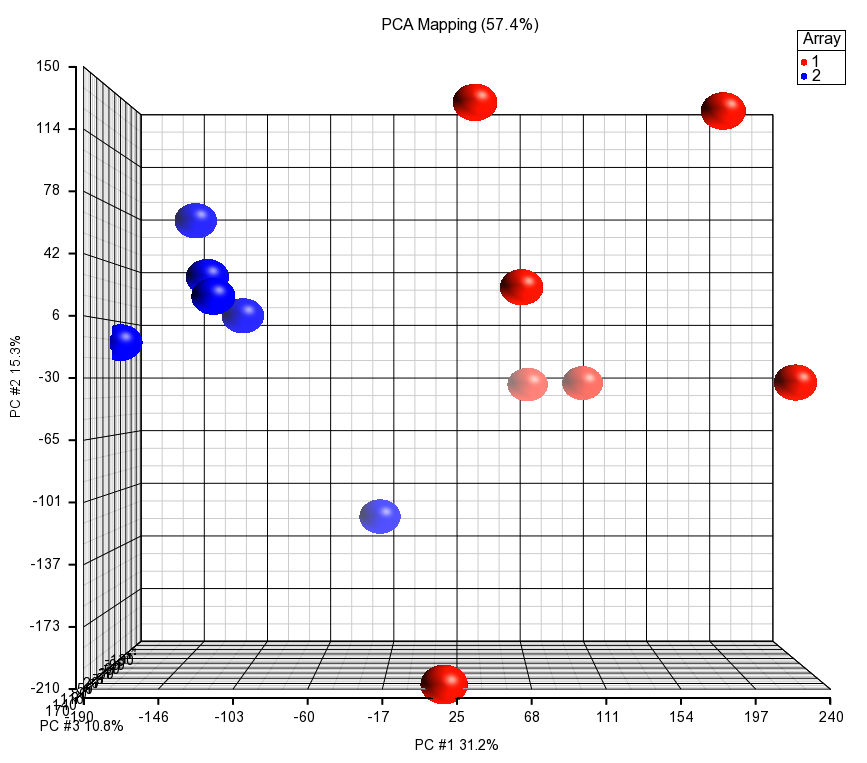

Supplement: Supplementary file 1 — Supplementary information [file 41598_2018_24505_MOESM1_ESM.doc]
